# On Some of the Relations between Chorea and Rheumatism

**Published:** 1892-09-03

**Authors:** 


### The Practitioner's Mirror and Retrospect.

[*The Editor will be glad to receive offers of co-operation and contributions from members of the profession. All letters should be addressed to* The Editor, The Lodge, Porchester Square, London, W.]

### MEDICINE.

### ON SOME OF THE RELATIONS BETWEEN CHOREA AND RHEUMATISM.

The cause of chorea has been constantly debated with very small results, but the frequency of its appearance along with, after or before, rheumatism is undeniable. It has been supposed by some that when a certain poison has entered the system it produces according to its surroundings the symptoms which we term chorea, acute rheumatism, endocarditis, tonsillitis, erythema nodosum, or other affections. The stress may fall sometimes on one organ, sometimes on another. The structure which to-day is capable of resisting an attack may in a year or two's time be the one which is disordered. When a new infection, or a revival of the activity of the poison occurs, the simple endocarditis of one occasion may be complicated by septic influences of another. Thus the whole string of complaints from erythema nodosum to malignant endocarditis may follow in turn a primary infection. There is indeed a certain likeness in the incidence and course of these diseases, their liability to recur, and their duration, besides the frequency with which they are found in the same individual, which lends some probability to the view. We find a similar relation in diseases produced by Fränkel's pneumococcus under varying circumstances. There is an organism which is frequently found in the mouths of healthy individuals, yet when it is introduced into the lungs under conditions which depress the resisting power of the individual, an attack of lobar pneumonia follows at once. Under other circumstances cerebro-spinal meningitis appears, with usually some concomitant pneumonia, and occasionally a more active virulence is developed and an infective form of one or other disease follows. But here we have a well-known cause, which can be readily isolated, cultivated by itself, and which is capable of reproducing the disease by inoculation. In the rheumatic group we have nothing of the kind, unless we accept Haigh's theory of the action of uric acid, which, however, seems to have little or no connection with chorea. Moreover, the diseases in question occur in such extremely different ways that in spite of their more or less frequent concurrence it seems impossible to suppose a common cause. It is quite certain that chorœa often follows a fright or some other strong mental emotion, and it is a pure assumption to imagine a latent rheumatism in such cases. Dr. T. W. Jenkins recently reported two cases of chorea where it appeared to be brought about by worms, and where it disappeared at once when they were killed. Indeed, it is more reasonable to regard chorea as a symptom produced by a variety of irritants acting on probably the cerebral cortex, and then to consider whether any one of the different types has an invariable connection with rheumatism. We have the mild chorea of Sydenham, and an athetoid variety: and from these Dr. L. C. Gray would distinguish chorea with rapid pulse and respiration, next violent chorea, that of pregnancy, the spasmodic or electric, and that accompanied by convulsions, with possibly the rare chorea of old age. To take only the ordinary chorea of Sydenham, which has a well marked *symptomen complexus* sufficient to entitle it to the position of a separate disease, it seems impossible to distinguish by any specific characters those cases which accompany rheumatism from those where there is no suspicion of it. Endocarditis occurs with both Sydenham's chorea and rheumatism, but it also follows pyæmia, so that we can hardly think it is solely due to a supposed rheumatic poison. Dr. Sturges seems to think that endocarditis from pure chorea is limited to the mitral valve, and does not tend to produce stenosis even there. Herringham noted, however, a presystolic murmur in one case. In forty-three out of eighty cases he could find no traces of rheumatism, and in six the chorea followed a mental shock within two days. In Dr. Garrod's eighty cases, reported at the same time, there were forty-four in which no previous attacks of rheumatism had occurred, but he believed that many were really rheumatic, since in childhood the signs of arthritis are usually slight and easily escape notice. He, too, mentioned a large number of fright cases, and in some statistics these have been said to amount to fifteen per cent. It may be argued that any depression renders the system apt for disease, just as pneumonia follows a blow or a fall, but we doubt whether there are many records of rheumatism following a fright, a beating, or a dreadful sight. Yet, these often precede a chorea indistinguishable in its course from that which accompanies rheumatism. Dr. Sturges, indeed, refers to an instance where fright was followed by chorea, and that again by rheumatism. Is it possible in such a case that the nerve disease lowered the nutrition of the joints which then fell a prey to the rheumatism poison? We often hear of the connection between joint pains and nerve disease. Is chorea the occasional sign of a local brain affection which more often manifests itself by arthritis? Such an explanation is perhaps quite as tenable as that rheumatism is the cause of chorea. Both Garrod and Herringham notice, we believe, a number of instances in which rheumatism followed instead of preceding chorea. Nevertheless, it is possible that more than one cause is capable of producing the central lesion resulting either in chorea or the malnutrition of joints from which rheumatism may arise. One fact is clear, that the two diseases are not by any means invariably manifested in the same person. In Germany and the United States the connection is much less often seen than it is here. Gowers thinks that the endocarditis which undoubtedly follows many pure attacks of chorea, is evidence of an altered blood state, and not an instance of malnutrition dependent on an altered state of the nervous system, or the result of muscular over exertion. Though his authority has great weight, there is no absolute proof of this blood change. The endocarditis does certainly follow and not precede the chorea in some instances, and may conceivably be produced through the nerve lesion. We cannot, on the whole, regard as proven the dependence of chorea and rheumatism upon a common poison. If they are connected at all, it is that they flourish in similar soils, otherwise chorea may as probably be the cause of rheumatism as the reverse.

